# vivaGen – a survival data set generator for software testing

**DOI:** 10.1186/s12859-020-3478-x

**Published:** 2020-04-29

**Authors:** Matthias Gietzelt, Christian Karmen, Petra Knaup-Gregori, Matthias Ganzinger

**Affiliations:** 1 Heidelberg University, Institute of Medical Biometry and Informatics, Im Neuenheimer Feld 130.3, Heidelberg, 69120 Germany; 2 Peter L. Reichertz Institute for Medical Informatics of TU Braunschweig and Hannover Medical School, Carl-Neuberg-Str. 1, Hannover, 30625 Germany

**Keywords:** Data set generator, Survival data, Biomarker, Java

## Abstract

An amendment to this paper has been published and can be accessed via the original article.

## Background

Software testing is an essential part of the software development process [1]. The testing process is usually divided into different consecutive test levels: function test (on function/method level), module test, integration test, and system test. The higher the test level, the higher is the need for more complex test data. Especially, medical and clinical systems require a systematic and exceptionally intensive testing process, before such systems can be considered to assist physicians in diagnosis or treatment of patients. Although these systems could and should be tested using real-world data, there are sometimes disadvantages for this approach: real-world data can possibly be influenced or interfered by effects such as confounders [2] or mediators [3]. These effects are non-random and in many cases unknown. As a consequence, they are difficult to control and must be taken into account when dealing with real-world data sets for testing purposes. In addition, real-world data might contain gaps, making some tests hard to perform.

The use of simulation data is often a very helpful way to test complex software systems at certain test stages. Simulated data can be designed in a way that all effects are well-defined and all relationships between attributes are controllable. Thus, for testing purposes it seems to be a sensible step to include test data from an environment that is fully controllable, but which is as close as possible to the intended setting into the test process.

### Simulation data in survival analysis

In a medical context, survival data typically emerge from longitudinal trials or registers, which include follow-up data. The main characteristics of survival data are the two related endpoints: the time period and the so-called event. The event can be for example “death” in case of overall survival observations, or “progression” in case of investigating progression-free survival periods. The time period is either the time, until the event happens, or otherwise, in case of no event, the duration of the observation period. If no event has occurred during the observation period, the observation is called right-censored [4].

Testing of systems that are processing survival data demands for well-defined data sets. It can be a challenging task to generate such survival data containing known effects. These effects should be found during the test by the system.

### Related work

Currently, there are a number of software packages available to simulate survival data in the statistical language *R* [5]: The *survsim* package [6] is able to generate single or multiple event data for different survival time distributions and it allows for a flexible handling of its parameters. However, it is not easy to define the characteristics of the attributes. For example, the number of subjects affected by an event is defined via corresponding distributions. This configuration might be no problem for experts, but is challenging for users with only basic experience.

The *genSurv* package [7] allows for the generation of data from a progressive illness-death model [8] using a variety of models: a Cox Markov model, a time-dependent Cox model, and a Cox Proportional Hazard model. Again, the configuration of effects of the covariates requires special skills.

There are other *R* packages, too, which provide survival data simulation functions. But the main focus of these packages is the provision of survival estimation functions rather than the simulation aspect itself. Thus, the simulation functions are provided as a test environment to test the core functions. For example, the package *splinesurv* [9] is able to simulate clustered survival data with a specified number of clusters in the sample. The simulated data is used to provide sample data of the correct format to test the main survival estimation functions of the package.

To the authors’ best knowledge, there is no package available that simulates data of a randomized controlled trial and supports a simple and comprehensive way for setting up survival time distribution parameters.

### Objective

The aim of this research was to design and to implement a data set generator for survival data that is comprehensively configurable and is also suitable for users with only basic experience in setting up survival time distribution parameters. The data set should simulate survival data of a typical randomized controlled trial with follow-up data and should be designed to feature defined differences between the randomized groups.

The simulated data set should contain both: random attributes, which are unrelated to the survival outcome, and attributes with defined and controllable effects. We define such non-random attributes as “biomarkers”, which are able to indicate, if a patient would be able to profit from a certain therapy in terms of an improved survival outcome. Such generated data sets can be used for testing in order to find the defined differences.

### Organization of the paper

The paper is organized as follows. Chapter “Implementation” contains information about the requirements of the survival data generation process, highlights the data organization and important points during the software development process, and describes how the requirements were met. “Results” contains information about the current status of the development. Finally, we describe the “Lessons learned” and our “Future plans” in the chapter “Conclusion”.

## Implementation

### Long-term survivors vs. short-term survivors

**Fig. 1:**
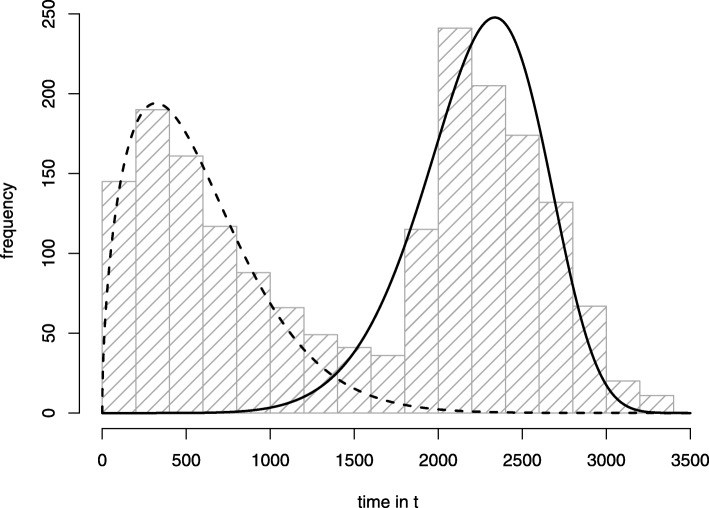
Survival time histogram.The figure shows a typical bimodal distribution of survival time in a longitudinal data set with fitted Weibull distributions at each peak. The fitted Weibull distributions were computed using a maximum likelihood estimation method. The dashed function characterizes the group of short-term survivors, the full line function the long-term survivors. The data used to plot the histogram originates from the “colon” data set, which was originally described in [10] and is provided as part of the *R* package *survival* [18]

In a typical longitudinal survival study, one may observe a bimodal distribution with two peaks in the survival time when represented in a histogram. Figure 1 shows a survival time histogram from the “colon” data set as an example for longitudinal survival data with two fitted Weibull distributions [10]. This data set shows a bimodal distribution. Indeed, this is not a result of an isolated study – a bimodal distribution of survival time is a common phenomenon [11]. As a consequence for modelling survival data, one has to consider two typical main groups of survivors: the so-called long-term survivors (LTS, group with a higher chance of a long-term survival) and the early decliners resp. short-term survivors (STS, group with a lower chance of a long-term survival). Therefore, the simulated cohort should represent such a LTS and a STS group.

### General cohort organization

One requirement for this data generator is to simulate survival data of typical randomized controlled trials on therapeutic comparisons with follow-up data. This means that the study cohort is organized in two balanced study arms: Arm B should be seen as the new therapy (e. g. a new medication), which is to be compared to the standard treatment of arm A (competing therapy/placebo arm). As a requirement, it should be possible to specify that one of both arms has a longer (or at least equal) survival compared to the other arm. This enables the specific manipulation of the data with an expected outcome in terms of an improved survival for a certain point in time *t* in one arm compared to the other arm. In the default settings, we chose arm B to be superior (or at least non-inferior) in comparison to arm A, i. e. *S*_*B*_(*t*)≥*S*_*A*_(*t*) for this scenario. But the user can also define *S*_*B*_(*t*)<*S*_*A*_(*t*), which means that arm A is superior compared to arm B.

**Fig. 2:**
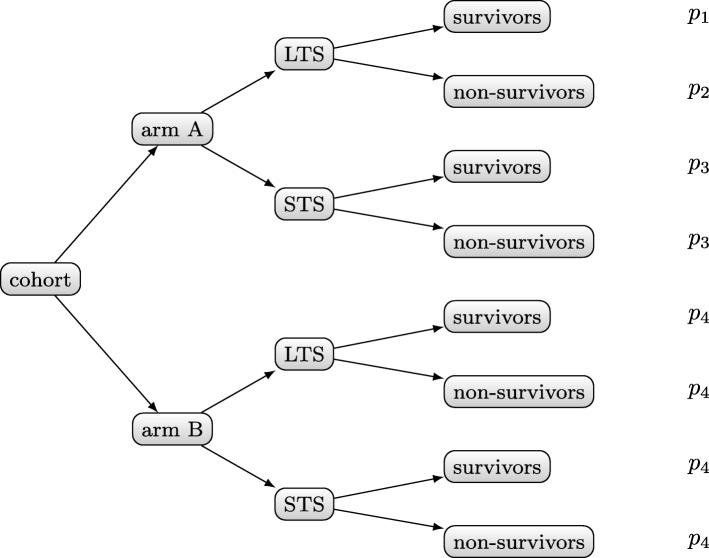
Organization of the cohort and study arms. The leafs of this tree have assignments of probabilities *p*_1_,…,*p*_4_ to indicate the prevalence of biomarkers in specific subgroups

After having derived the necessity for distinguishing between the LTS and the STS group, a generalized model for generating the cohort data can be drawn. As shown by the example of Fig. 2, the cohort will be organized into two arms, both containing LTS and STS. Thereby, our approach specifies survivors and non-survivors in order to model defined effects into the data.

### Setting up survival time distribution parameters

Another requirement is to have a convenient way to set up abstract survival time distribution parameters, which can be difficult to adjust. Since practitioners have an intuition about the estimation of the survival rate *S*(*t*) for a certain point in time *t*, we decided to use this measure for setting up the survival time distribution. Thus, the user will be able to configure the survival function in a way that it touches a specific point in a survival plot.

In contrast to other approaches, this data generation approach should allow for the survival time distribution parametrization of a specific survival rate at a certain point in time. In the following paragraphs we introduce our concept for a simple parametrizations for a Weibull- and a log-logistic-distributed random variable for modelling survival time.

Let *Y*_Wei_∼ Wei(*λ*,*k*) be a Weibull-distributed random variable with *λ* as a scale parameter and *k* as a shape parameter and $\lambda, k \in \mathbb {R}_{> 0}$. Given a point in time $t \in \mathbb {R}_{> 0}$ with a fixed corresponding survival probability *S*(*t*)∈(0,1), the survival function *S* of the Weibull distribution can be solved for the unknown parameter *λ*:

1 $$ S(t) = e^{-\left(\frac{t}{\lambda} \right)^{k}}.  $$

It emerges for the parameter *λ*, which depends on *t*, *S*(*t*) and *k*:

2 $$ \lambda = 10^{\log \left(t \right) - \log \left(-\ln \left(S(t) \right) \right) \cdot k^{-1}}.   $$

With this result we are able to estimate parameter *λ*, but not *k*, which models the shape of the survival function. It seems that there is no useful possibility to estimate this parameter in a convenient way, so that the user still has to choose the shape parameter explicitly.

The existence of *λ* is shown in the following term by analyzing the exponent of the right hand side of Eq. 2:

3 $$ \log \underbrace{\left(t \right)}_{>0} - \log \underbrace{\left(-\overbrace{\ln \underbrace{\left(S(t) \right)}_{>0}}^{<0 \Rightarrow S(t)<1} \right)}_{>0} \; \cdot k^{-1}.  $$

It emerges that

1. *t*>0
2. *k*≠0, but the Weibull distribution already demands for *k*>0
3. 0<*S*(*t*)<1
4. *λ*>0 (as follows from Eq. 2).

These results show that no additional limitations for the parameters must be met for the Weibull distribution.

Let *Y*_LL_∼ LL(*α*,*β*) be a log-logistic-distributed random variable with $\alpha, \beta \in \mathbb {R}_{> 0}$. Given a point in time $t \in \mathbb {R}_{> 0}$ with a fixed corresponding survival probability *S*(*t*)∈(0,1), the survival function *S* of the log-logistic distribution

4 $$ S \left(t \right) = \frac{1}{1 + \left(\frac{t}{\alpha} \right)^{\beta}}  $$

can be solved for the unknown parameter *α*:

5 $$ \alpha = t \cdot 10^{-\log \left(S^{-1}(t) - 1 \right) \cdot \beta^{-1}}.   $$

The existence of *α* is shown in the following term by investigating the exponent of the right hand side of Eq. 5:

6 $$ \log \left(\underbrace{S^{-1}(t) - 1}_{>0 \Rightarrow 0<S(t)<1} \right) \cdot \beta^{-1}.  $$

It emerges that

1. *t*>0
2. *β*≠0, but the log-logistic distribution already demands for *β*>0
3. 0<*S*(*t*)<1
4. *α*>0 (as follows from Eq. 5) for *t*>0.

These results show that no additional limitations for the parameters must be met for the log-logistic distribution. *β* has a similar influence as compared to *k* of the Weibull distribution.

With the help of Eq. 2 (and Eq. 5, resp.), it is possible to easily set up survival time distribution parameters.

### Independent random and non-random attributes

The generated independent attributes should represent both: random and non-random attributes. The non-random attributes are called “biomarker” in this paper. Biomarkers should be able to indicate, whether a patient will be responsive with regard to a certain treatment, or not. The requirement for the generation process was to have at least one nominal and one numeric biomarker. Biomarkers should have a configurable intensity. For the generation process it is mandatory that the biomarkers are overall equally distributed in study arms A and B, but show an accumulation in specific subgroups.

## Results

**Fig. 3:**
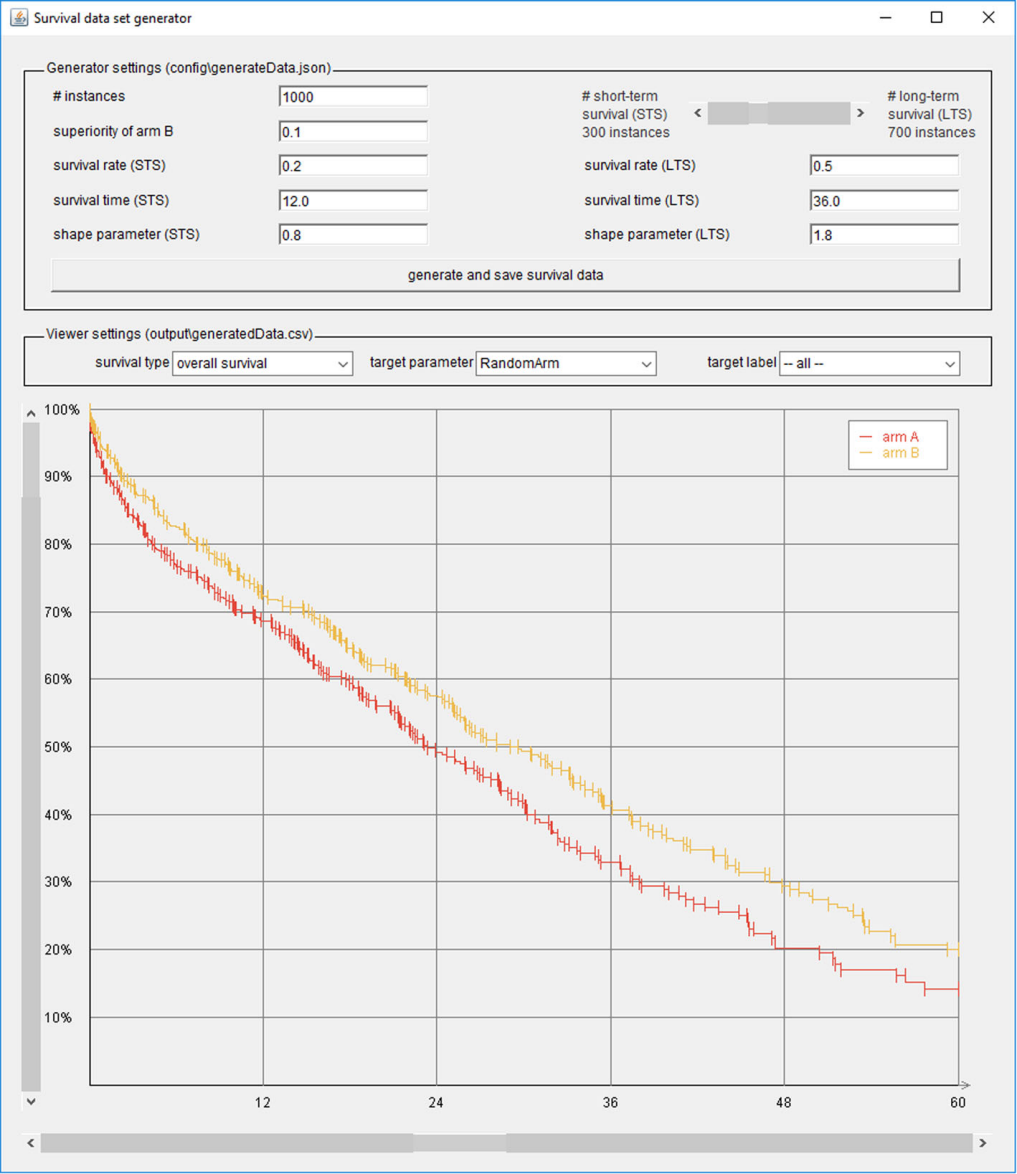
Screenshot of the GUI of *vivaGen*

The name of the software presented in this paper is *vivaGen*. Currently, in *vivaGen* it is possible to configure the above mentioned features in a graphical user interface (GUI). The GUI also provides an output visualization in terms of a survival plot. This plot instantly shows the changes made. Figure 3 shows a screenshot of the GUI. A more extensive configuration can be made using a configuration file in JavaScript Object Notation (JSON) format [12]. All parameters related to data generation presented in this section can be set in this file, too.

### Independent random and non-random attributes

Random attributes are being generated using the transformations given in the *Background* section. Currently, the following distributions are supported:

- Uniform distribution (nominal and numeric attributes are generated)
- Binomial distribution
- (Standard-)normal distribution
- Exponential distribution
- Weibull distribution
- Log-logistic distribution

*vivaGen* also allows for the definition of two kinds of biomarkers for each therapy arm independently: one nominal and one numerical biomarker. In the current state of development, the nominal biomarker is binomially distributed and the numerical biomarker follows a normal distribution. The specification of the biomarkers in this section will reflect the definition of those of arm A as an example, but the biomarkers of arm B are specified respectively.

For the biomarker generation process we make use of the cohort organization described in the *Implementation* section. The nominal biomarker is actually a composite attribute of four binomial distributions Bin(*n*_*i*_,*p*_*i*_), which have four different probability values *p*_*i*_ in order to emphasize the ability to survive for particular subgroups. Binomial distributions provide results in terms of “success” and “failure”, i. e. “true” and “false”. Figure 2 shows the assignments of the probability values *p*_1_,…,*p*_4_ as annotations of the leafs.

The assignment of probability values leads to a selective emphasizing of particular subgroups. The distinction within the LTS group of arm A was done to better emphasize the difference between both subgroups. Tests confirmed that a differentiation of the LTS group is necessary for the nominal attribute in order to get a distinct and visible result – especially for the arm that is inferior to the other. Because of the inferiority of the results of this arm as a whole, improvements might be only very slight, when modelling the LTS survivor group alone.

For configuring the biomarker’s intensity, the user has to choose the probability values of

7 $$\begin{array}{*{20}l} p_{1} & = p(\text{biomarker} \vert \textrm{arm A} \cap \textrm{LTS} \cap \text{survivors}), \end{array} $$

8 $$\begin{array}{*{20}l} p_{2} & = p(\text{biomarker} \vert \textrm{arm A} \cap \textrm{LTS} \cap \textrm{non-survivors})\ \text{and} \end{array} $$

9 $$\begin{array}{*{20}l} p_{4} & = p(\text{biomarker} \vert \textrm{arm B}) = p(\text{biomarker}) = p_{\text{prevalence}}. \end{array} $$

The probability *p*_4_ is equal to the overall prevalence of the biomarker. During the development process, it seemed to be reasonable that practitioners have an intuition about it. The missing probability *p*_3_=*p*(biomarker|arm A∩STS) can be computed based on the other probabilities in order to meet the requirement that the biomarker is overall equally distributed in study arm A and B. Overall equally distributed in this sense means that the number of values for “true” is the same in arm A and arm B.

The numerical biomarker is specified as a composition of two normally distributed variables. The next equations define the identifiers of the distribution parameters:

10 $$\begin{array}{*{20}l} \mathrm{B}_{\textrm{biomarker, num}} & \sim & \mathcal{N}\left(\mu_{\text{biomarker}}, \sigma_{\text{biomarker}}^{2} \right) \text{and} \end{array} $$

11 $$\begin{array}{*{20}l} \mathrm{B}_{\textrm{non-biomarker num}} & \sim & \mathcal{N}\left(\mu_{\textrm{non-biomarker}}, \sigma_{\textrm{non-biomarker}}^{2} \right), \end{array} $$

whereat the term “biomarker” is used for indicating that the biomarker might be present and “non-biomarker” that the biomarker is not necessarily present, because it is very likely that both distributions do overlap. The grade of overlapping can be interpreted as one measure of the configurable intensity of this biomarker.

Since the numerical biomarker should also have the same frequency in both arms, we applied a two-step procedure for assigning the two normally distributed variables. First, we used to same strategy as for the nominal biomarker for indicating the same number of subjects in arm A and arm B as “true” for having the biomarker. Furthermore, the intensity of the biomarker can be adjusted with similar, but independent adjustment parameters as for the nominal attribute. After that, values of B_biomarker, num_ are assigned to those subjects marked as “true” and B_non-biomarker num_ to those marked as “false”.

### Long-term survivors vs. short-term survivors

In both groups there are subjects, who have survived and others who have not survived. A simple histogram of survival time is only able to represent the observation period of the subjects. There is no indication about the subjects’ survival status. In other words, the histogram does not reflect whether the subjects

- have refused to participate in the study or
- have been rejected from study observation (e. g. because of non-adherence) with status “alive” or
- died during the observation period.

**Fig. 4:**
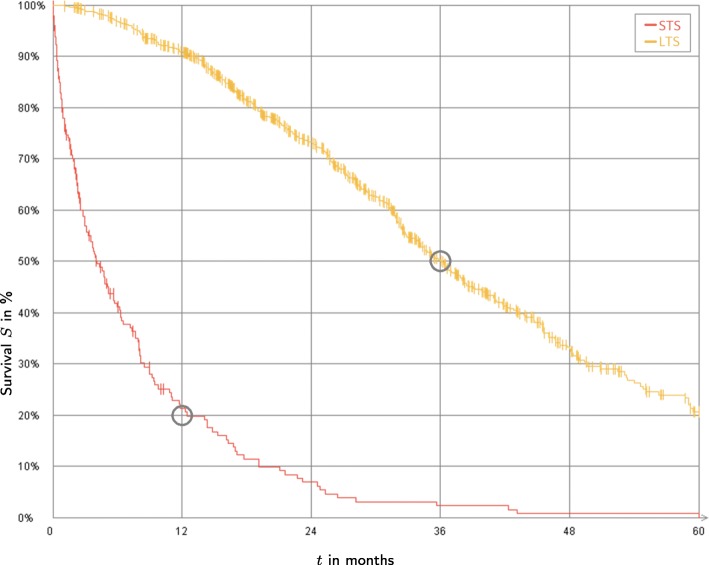
LTS vs. STS. Each survival curve touches a specified point: The black curve (in color reproduction: red) of STS touches (*t*, *S*(*t*)) = (12 months, 20 %) and the light gray curve (in color reproduction: yellow) of LTS touches (*t*, *S*(*t*)) = (36 months, 50 %)

Survival plots are diagrams, which are able to visualize outcomes for survival time and survival status. One example for such a survival plot is the Kaplan-Meier survival curve. Figure 4 illustrates such a survival plot. Using the results of the section *setting up survival time distribution parameters*, the diagram shows an example configuration of a resulting LTS and STS curve. Both curves are touching specified points. If there is no need for distinguishing between the groups LTS and STS, the size of one of the groups can be set to 0.

### Nominalization of attributes

In order to visualize numeric attributes (random as well as non-random attributes) in a survival plot, these attributes have to be nominalized. Therefore, a nominalization process will be applied that uses a cutoff value *c* for dichotomizing attribute *A* into two survival functions: *S*(*t*|*A*≤*c*) and *S*(*t*|*A*>*c*). The cutoff value *c* will be computed in order to maximize the area between the emerging survival functions in a survival plot to get the best discriminating value. The time complexity for the nominalization process is in $\mathcal {O} \left (N^{2} \right)$, whereas *N* denotes the number of subjects.

We evaluated the nominalization process using the following hardware and software equipment: Intel CPU i5-4570@3.20 GHz with 16 GByte RAM, Java SE Runtime Environment (build 1.8.0_131-b11) on a Microsoft Windows 7 Professional 64-bit version 6.1.7601 Service Pack 1 Build 7601. Table 1 shows the computing times for nominalizing a single numeric attribute for different cohort sizes.

**Table 1:** Computing times for nominalization

Number of subjects *N*	Computing time for nominalization in *s*
100	0.025
500	0.392
1000	1.353
5000	31.90
10000	142.6

The computing times for nominalization (in seconds) are given per attribute to nominalize

### Generation of single events

For our use case, it was adequate that the endpoint of interest is a single event such as overall survival or progression-free survival. Thus, in the current state of the system, we only consider single event outcomes.

## Conclusion

In this paper we introduced the software *vivaGen* for flexibly and comprehensively generating simulated survival data sets of a typical randomized controlled trial with follow-up data. *vivaGen* is designed to feature defined differences between the randomized groups. The data sets include random and non-random attributes and can be used for testing software for predicting or modelling survival outcomes. The non-random attributes are of configurable intensity regarding survival outcomes. The GUI of *vivaGen* allows for a targeted specification of survival parameters and attributes, which can be visualized and controlled using survival plots. The generated survival data can have a bimodal distribution of survival time in order to simulate long-term and short-term survivors.

### Lessons learned

The main question during the implementation process of *vivaGen* was how to achieve the equal distribution in arm A and arm B of the biomarkers. We tried many different approaches until we got the final solution presented in this paper. First implementations showed a low intensity, especially of the nominal biomarker of the arm that is inferior compared to the other.

The requirement of modelling one arm as being superior compared to the other was another challenge. We handled the problem with a simple solution and corrected the survival rate of each subgroup (see Fig. 2). The solution was to add half of the correction factor to the survival rate of arm B and subtract the other half from the survival rate of arm A. Thereby, one has to consider the sizes of the single subgroups.

In recent studies, also concurrent endpoints are observed [13]. These endpoints can be, for example, progression of disease or death. In the current state of development of *vivaGen*, a combination of such endpoints has not been implemented yet. But the authors consider the generation of multiple events, which can be interpreted as either different concurrent events or recurrent events of the same type.

We used data sets generated by *vivaGen* to test a research decision support system (DSS) developed by our group. Aim of the DSS is to give a recommendation for a certain therapy for a new patient given a case base of patients and known survival outcomes. The defined relations of attributes and survival outcome in the generated data sets were successfully used to train and test the DSS on system level.

### Future plans

First, the survival data set generator currently generates independently distributed random attributes. One of the next steps is to generate these attributes with configurable correlations between them. This could be achieved by implementing an attribute representing a certain “degree of dependence” between other attributes and the outcome attributes (survival time and survival status). Bender et al. describe techniques and models to generate simulation data regarding outcomes of the Cox proportional hazards model [14].

Second, currently *vivaGen* only supports the generation of binomial and normal distributions for the biomarkers. Other distributions could be added to gain more flexibility for the data generator.

Third, the parametrization of the generated biomarkers could be made more user-friendly. The idea is to provide one value of intensity for each biomarker, for example in the interval [0,1] such that 1 stands for a maximum possible intensity and 0 for no intensity.

Fourth, the GUI could be extended to configure all attributes in a more user-friendly environment. This also includes the visualization of histograms for configuring the random and non-random attributes.

Fifth, multiple events could be supported by the generator, such as recurrent events to support the test of systems demanding for such complex outcome data.

These future plans are intended for intensifying the tests of our DSS and should also encourage a broader spectrum of researchers as well as software developers to generate survival simulation data using *vivaGen* for testing their software.

## Availability and requirements

**Project name**: *vivaGen*

**Project home page**: https://sourceforge.net/projects/vivagen/

**Operating system(s)**: Platform independent

**Programming language**: Java

**Other requirements**: Java SE Runtime Environment 8 or higher, WEKA package [15], and a JSON package [12] with a suitable and compatible license, such as the JSON Software Bundle provided by Oracle [16].

**License**: GPL v2 [17]

**Any restrictions to use by non-academics**: no

## Data Availability

All data and software used and developed for this research are available at https://sourceforge.net/projects/vivagen/.

## Abbreviations

DSS: Decision support system
GUI: Graphical user interface
JSON: JavaScript object notation
LTS: Long-term survivor
STS: Short-term survivor
